# Supplementary material for: Effect of blood analysis and immune function on the prognosis of patients with COVID-19
Source: PLoS One. 2020 Oct 30;15(10):e0240751. doi: 10.1371/journal.pone.0240751 (PMC7598452; doi:10.1371/journal.pone.0240751)
Supplement: S1 File — (DOCX) [file pone.0240751.s002.docx]

STROBE Statement—checklist of items that should be included in reports of observational studies

|  | Item No. | Recommendation | Page  No. | Relevant text from manuscript |
| --- | --- | --- | --- | --- |
| **Title and abstract** | 1 | (*a*) Indicate the study’s design with a commonly used term in the title or the abstract | 2 | Abstract. This retrospective study investigated …… |
|  |  | (*b*) Provide in the abstract an informative and balanced summary of what was done and what was found | 2 | The information was described in the abstract. |
| Introduction | | | |  |
| Background/rationale | 2 | Explain the scientific background and rationale for the investigation being reported | 4-5 | The introduction explains them. |
| Objectives | 3 | State specific objectives, including any prespecified hypotheses | 5 | The aim of this study is given. |
| Methods | | | |  |
| Study design | 4 | Present key elements of study design early in the paper | 6 | They were introduced in study design. |
| Setting | 5 | Describe the setting, locations, and relevant dates, including periods of recruitment, exposure, follow-up, and data collection | 5-6 | The study population comprised patients with COVID-19 pneumonia, who had been admitted to the ward of the Third Batch of Chongqing Medical Aid Team in Wuhan city of Hubei province in China, from 4 February 2020 to 16 February 2020. Relevant information was described in Materials and methods. |
| Participants | 6 | (*a*) *Cohort study*—Give the eligibility criteria, and the sources and methods of selection of participants. Describe methods of follow-up  *Case-control study*—Give the eligibility criteria, and the sources and methods of case ascertainment and control selection. Give the rationale for the choice of cases and controls  *Cross-sectional study*—Give the eligibility criteria, and the sources and methods of selection of participants | 6 | The patients in this study were confirmed to have SARS-CoV-2 infection by real-time reverse transcription polymerase chain reaction (RT-PCR) assay, from nose and throat swab samples. At least one ground glass change in the lung was indicated on chest computed tomography (CT) scan. A blood routine and immune function test was completed within 12 hours after admission. The primary outcome was in-hospital death. All survivors discharge before 1 April 2020. |
|  |  | (*b*) *Cohort study*—For matched studies, give matching criteria and number of exposed and unexposed  *Case-control study*—For matched studies, give matching criteria and the number of controls per case |  |  |
| Variables | 7 | Clearly define all outcomes, exposures, predictors, potential confounders, and effect modifiers. Give diagnostic criteria, if applicable | 6 | The primary outcome was in-hospital death. The relevant information was given in materials and methods. |
| Data sources/ measurement | 8* | For each variable of interest, give sources of data and details of methods of assessment (measurement). Describe comparability of assessment methods if there is more than one group | *5-6* | The data was collected from clinical data of patients with COVID-19. |
| Bias | 9 | Describe any efforts to address potential sources of bias |  |  |
| Study size | 10 | Explain how the study size was arrived at | 6 | The study population comprised patients with COVID-19 pneumonia, who had been admitted to the ward of the Third Batch of Chongqing Medical Aid Team in Wuhan city of Hubei province in China, from 4 February 2020 to 16 February 2020. |

Continued on next page

| Quantitative variables | 11 | Explain how quantitative variables were handled in the analyses. If applicable, describe which groupings were chosen and why | 7 | Data were analyzed using SPSS 21.0 (SPSS Inc. Chicago, IL, USA). Continuous variables are shown as median (interquartile range or IQR), and categorical data as percentage. |
| --- | --- | --- | --- | --- |
| Statistical methods | 12 | (*a*) Describe all statistical methods, including those used to control for confounding | 7 | Statistical methods included: the Mann-Whitney U test, the chi-squared test, ROC curves, univariate and multivariate logistic regression analysis. |
|  |  | (*b*) Describe any methods used to examine subgroups and interactions | 7 | All factors with P ≤ 0.25 in the univariate logistic regression model were included in the multivariate model. And the interactions of some factors were analysed. |
|  |  | (*c*) Explain how missing data were addressed | 7 | Showed in Statistical analysis section. |
|  |  | (*d*) *Cohort study*—If applicable, explain how loss to follow-up was addressed  *Case-control study*—If applicable, explain how matching of cases and controls was addressed  *Cross-sectional study*—If applicable, describe analytical methods taking account of sampling strategy | 7 | Showed in Statistical analysis section. |
|  |  | (*e*) Describe any sensitivity analyses | 7 | Showed in Statistical analysis section. |
| Results | | | | |
| Participants | 13* | (a) Report numbers of individuals at each stage of study—eg numbers potentially eligible, examined for eligibility, confirmed eligible, included in the study, completing follow-up, and analysed | 7-8 | Overall, 93 persons were treated during the study period, 85 (49 men) of whom met the inclusion criteria for this study. Seventy-one patients survived to discharge, while 14 patients died in hospital. |
|  |  | (b) Give reasons for non-participation at each stage | 7 | Six patients were excluded due to absence of specific blood test within 12 hours after admission or negative SARS-CoV-2 result of nasopharyngeal swab sample. One patient in his 90s died of old age, although the condition of COVID-19 was obviously improved after treatment. |
|  |  | (c) Consider use of a flow diagram |  | No of applicable |
| Descriptive data | 14* | (a) Give characteristics of study participants (eg demographic, clinical, social) and information on exposures and potential confounders | 8-9 | Relevant information was showed in tables. |
|  |  | (b) Indicate number of participants with missing data for each variable of interest | 8 |  |
|  |  | (c) *Cohort study*—Summarise follow-up time (eg, average and total amount) | 8 | Seventy-one patients survived to discharge, while 14 patients died in hospital. |
| Outcome data | 15* | *Cohort study*—Report numbers of outcome events or summary measures over time | 8 | Seventy-one patients survived to discharge, while 14 patients died in hospital.  The remaining hospitalized survivors (n=67) underwent blood tests, 2 weeks after treatment (Table 7).  In 53 patients who tested cellular immunity of the survival group after 2 weeks of hospitalization, the count of lymphocyte subsets returned to normal range in most patients (Table 8). |
|  |  | *Case-control study—*Report numbers in each exposure category, or summary measures of exposure |  |  |
|  |  | *Cross-sectional study—*Report numbers of outcome events or summary measures |  |  |
| Main results | 16 | (*a*) Give unadjusted estimates and, if applicable, confounder-adjusted estimates and their precision (eg, 95% confidence interval). Make clear which confounders were adjusted for and why they were included | 7-14 | Relevant statistical information was showed in the tables of result section. |
|  |  | (*b*) Report category boundaries when continuous variables were categorized | 7-14 | Clearly showed in the tables. |
|  |  | (*c*) If relevant, consider translating estimates of relative risk into absolute risk for a meaningful time period |  |  |

Continued on next page

| Other analyses | 17 | Report other analyses done—eg analyses of subgroups and interactions, and sensitivity analyses |  |  |
| --- | --- | --- | --- | --- |
| Discussion | | | | |
| Key results | 18 | Summarise key results with reference to study objectives | 14-19 | Key results were clearly summarised and discussed the results with previous studies in the discussion section. |
| Limitations | 19 | Discuss limitations of the study, taking into account sources of potential bias or imprecision. Discuss both direction and magnitude of any potential bias | 19-20 | Relevant limitations of the study were discussed. |
| Interpretation | 20 | Give a cautious overall interpretation of results considering objectives, limitations, multiplicity of analyses, results from similar studies, and other relevant evidence | 15-20 | A cautious overall interpretation was given in discussion section. |
| Generalisability | 21 | Discuss the generalisability (external validity) of the study results | 21 | Described in conclusion section. |
| Other information | |  | | |
| Funding | 22 | Give the source of funding and the role of the funders for the present study and, if applicable, for the original study on which the present article is based | 1 | This project was supported by the Medjaden Academy & Research Foundation for Young Scientists (Grant No. COVID-19-MJA20200323) and the clinical research project of COVID-19 in Chongqing Medical University (Grant No. 0064). |

*Give information separately for cases and controls in case-control studies and, if applicable, for exposed and unexposed groups in cohort and cross-sectional studies.

**Note:** An Explanation and Elaboration article discusses each checklist item and gives methodological background and published examples of transparent reporting. The STROBE checklist is best used in conjunction with this article (freely available on the Web sites of PLoS Medicine at http://www.plosmedicine.org/, Annals of Internal Medicine at http://www.annals.org/, and Epidemiology at http://www.epidem.com/). Information on the STROBE Initiative is available at www.strobe-statement.org.
